# Food Promotion and Children's Health: Considering Best Practices for Teaching and Evaluating Media Literacy on Food Marketing

**DOI:** 10.3389/fpubh.2022.929473

**Published:** 2022-07-11

**Authors:** Charlene Elliott, Emily Truman, Michelle R. Nelson, Cyndy Scheibe, Liselot Hudders, Steffi De Jans, Kara Brisson-Boivin, Samantha McAleese, Matthew Johnson, Lauren Walker, Kirsten Ellison

**Affiliations:** ^1^Department of Communication, Media and Film, University of Calgary, Calgary, AB, Canada; ^2^Department of Advertising, College of Media, University of Illinois Urbana-Champaign, Urbana, IL, United States; ^3^Psychology, Ithaca College, Ithaca, NY, United States; ^4^Department of Communication Sciences, Ghent University, Ghent, Belgium; ^5^MediaSmarts, Ottawa, ON, Canada; ^6^APPLE Schools, Edmonton, AB, Canada

**Keywords:** media literacy, food literacy, food marketing, children, evaluation, health

## Abstract

Food marketing to children is ubiquitous and persuasive. It primarily promotes foods of poor nutritional quality, influences children's food preferences and habits, and is a factor in childhood obesity. Given that food marketing relentlessly targets children in traditional/digital media and the built environment, children need critical media literacy skills that build their understanding of food marketing's persuasive effects. However, little research connects media literacy with food marketing and health, including effective strategies for teaching and evaluating such programming for children. This perspective presents the outcomes of a stakeholder meeting on best practices in *teaching* and *evaluation* on media literacy and food marketing to children. Strategies for promoting critical thinking (teaching content, teaching practices, teaching supports, and parent/caregiver involvement), and strategies for measuring critical thinking (program effectiveness and broader long-term impacts) were identified. These include, among other things, the need to capture the *range of marketing formats* and *current food promotion trends*, to include *inquiry-based* and *co-creation activities*, and to support *ongoing media literacy development*. Overall, these strategies suggest useful criteria for media literacy programming related to food marketing, and highlight the importance of media literacy for giving children the skills to navigate a complex food environment.

## Introduction

Food marketing to children is a “pervasive and persuasive” problem worldwide (1). It primarily promotes foods of poor nutritional quality, influences children's food attitudes, preferences and habits, and has been linked to childhood obesity (1, 2). In Canada, children are relentlessly targeted with food marketing appeals, encountering 8–10 food and beverage advertisements a day on television, 30 food ads a week on social media, and an estimated 25 million food advertisements a year online (3, 4). Canadian children also routinely see food marketing in and around schools, encountering a median of 18 food advertisements within 400 metres of schools in urban settings (5, 6). Despite this pervasive food marketing, “food literacy” programs for children rarely include training in critical media literacy–a skill necessary to help them understand and evaluate food marketing's persuasive effects. And while some media literacy programs related to food exist, limited scholarship connects media literacy with food marketing and health, resulting in a lack of frameworks for evaluating such programs.

In light of this, we convened a Best Brains Exchange (BBE) that connected international experts and stakeholders in both media literacy and food/health to share insights into research and practice, and to identify best practices in teaching and evaluation in this area. Our perspective summarises the meeting results, offering a snapshot of critical media literacy “best practices” as a starting point to formulate new thinking and evaluative frameworks for food-focused media literacy programming.

Food literacy is an extremely popular term and topic, used to describe the idea of proficiency in food-related skills and knowledge. In Canada, various child-targeted food literacy programs exist, ranging from the *Rainbow Plate* (sensory experience of food),^1^ *Growing Chefs* (cooking/educational programming)^2^ and Six *by Sixteen* (nutrition/cooking programming).^3^ The focus of such programming is primarily on the acquisition of functional skills (e.g., food preparation, cooking). Accordingly, the evaluation and perceived “success” of food literacy programming focuses on whether these skills have been acquired and on nutrition outcomes (7, 8).

Media literacy is different. While media literacy also addresses functional skills (e.g., how to program a computer, how to create a website), it is equally, and fundamentally, about critical skills. Media literacy is “the ability to access, analyse and evaluate messages across a variety of contexts” (9), and to put these skills into meaningful action (10). Media literacy is essential when it comes to understanding food marketing, as it promotes constructs that are not core to “food literacy” or “health literacy” (11). In particular, media literacy facilitates the *examination and interpretation of the effects of persuasive communication techniques on the “meaning” and desirability of foods–and also on perceptions of nutrition*. Media literacy also facilitates *understanding of food marketing's persuasive “power” or the specific techniques used to persuade* (12), enabling understanding of visual/linguistic elements of food marketing (e.g., spokes-characters, colours, design).

Although questions of evaluation are commonplace in food literacy research, they (as noted above) focus primarily on assessing functional skills, sidestepping the critical skills needed to assess food marketing messages. Our BBE meeting connected international experts, practitioners and trainees to share knowledge and establish methods to evaluate critical media literacy and food programming. Following knowledge exchange (on research projects and practitioner programing), stakeholders participated in group discussions to identify best practices in teaching critical media literacy content on food marketing, and strategies for evaluating such interventions.

## Stakeholder Meeting Results

Meeting content and discussion generally fell into two main categories: strategies for *promoting* and strategies for *measuring* critical media literacy skills. Table 1 summarises these categories and their subcategories.

**Table 1:** Strategies for promoting and measuring critical thinking in intervention programming.

	**Area**	**Strategies**
Promoting critical thinking	Content for children	• Use a co-creation method with different stakeholders to design effective interventions • Ensure lessons are flexible and reflect the changing media environment • Prioritise the ability to recognise persuasive intent across a range of mediated messages and formats • Include real-world examples, and allow children to be the content experts
	Teaching practices	• Integrate critical media literacy into existing subject-area lessons across all grades (from K-12) • Use a key concepts approach • Design inquiry-based lessons • Incorporate hands-on learning activities • Provide children with the opportunity to make media • Acknowledge children's positive feelings towards brands and food products • Acknowledge the instructor's own vulnerability to media • Foster an environment of critical respect • Encourage scepticism, not cynicism • Encourage action and engaged citizenship
	Teaching support	• Support and facilitate professional development and training on media literacy/critical thinking • Create curriculum-linked lessons-in-a-box (tool-kits; lesson plans) • Encourage peer training opportunities
	Parent/caregiver involvement	• Engage parents/caregivers in media literacy activities • Provide support to increase parental/caregiver media literacy and facilitate professional development and training on media literacy/critical thinking
Measuring critical thinking	Program effectiveness	• Use a mixed methods approach • Use quantitative methods (i.e., pre/post-test) for measuring recognition and understanding (changes in knowledge levels) • Use qualitative methods to explore analysis and evaluation skills (changes in perceptions/attitudes)
	Broader/longer-term impacts	• Track reach of program (i.e., number of children exposed) • Use post-post-evaluations to gauge learning over longer term • Measure capabilities and confidence of the children's community of trusted adults to support the development and application of critical media literacy skills
Resources	• **Media Literacy & Food Marketing (MLFM) Lesson Plans and Tool-Kits** (https://www.ucalgary.ca/food-marketing/knowledge-translation, includes lessons on persuasive elements of food packaging, interactive tool-kits, fact sheets for parents/caregivers). • **Making Media for a Healthier U: Nutrition and Advertising Literacy** (https://makingmedia.media.illinois.edu/curriculum/waukegan-pilot-nutrition-and-advertising-curriculum-may-2014/, includes lessons on nutrition and advertising literacy, student worksheets, teacher guide). • **Project Look Sharp** (www.projectlooksharp.org, includes lessons on health and consumer education, and professional development resources for teachers). • **ReclameWijs** [Advertising-Wise] (https://reclamewijs.ugent.be/, includes interactive media literacy games and vlog content around brand awareness). • **MediaSmarts** (www.mediasmarts.ca/marketing-consumerism/resources-teachers, includes lessons on food advertising, the nutritional value of foods advertised on television/magazines, and an interactive unit on food marketing on the web) • **Apple Schools** (www.appleschools.ca/monthly-campaigns-march, includes teacher materials on nutrition and food advertising).	

## Discussion

Strategies generated provide useful criteria for media literacy programming on food marketing, both in terms of *promoting* and *evaluating* critical thinking in children. When it comes to best practices for teaching media literacy, beyond the obvious need to ensure the content is tailored to the age cohort being taught, four key recommendations emerged.

First, it is critical to draw children's attention to the *range of marketing formats* (e.g., influencer marketing, advertising banners, YouTube pre-rolls, in-game advertising, advergames, packaging, sponsorships) and *food media* that promote food and beverage products to them. Given the diverse advertising formats available (especially within digital media), some marketing formats may be difficult for children to identify without explicit training. Second, one must be nimble when selecting specific examples of food marketing: examples must *capture current food promotion trends* to be relevant to children's lives and connected to the world outside of the classroom. Third, include *inquiry-based activities* that teach children how to become agents and co-creators of their own media investigations. For example, after teaching children how to distinguish marketing claims from factual claims on a food package, or after exploring how colours (such as green or brown) subtly persuade consumers about the overall healthfulness of packaged foods, then give them actual food products to analyse or sort (e.g., healthy/less healthy). Inquiry-based activities are fundamental to children's ability to identify, understand, and evaluate media content, particularly as it relates to persuasive intent, and encourage children to *transfer their skills into meaningful action*. Challenge and game-based learning can also help heighten children's motivation to critically reflect on media content (13). Fourth, *use media creation activities* to prompt children's critical thinking with respect to food marketing messages. Ask children to draw from the persuasive techniques identified to create their own promotional messages for fruit or vegetables (thus applying marketing techniques to healthy eating), or to create a “subvertisement”–a parody ad that functions as a form of critique [see (14)]. Such media creation activities fuse creative and critical activities, encouraging children to apply media literacy to positive outcomes. Finally, part of “best practices” when it comes to teaching media literacy and food marketing entails increased *teaching and parental/caregiver supports*. This is necessary because nutrition knowledge and the ability to navigate food marketing appeals are highly variable among teachers and parents/caregivers. For teachers, such support includes professional development/training, as well as the creation of ready-to-use evidence-based resources (i.e., lessons-in-a-box) clearly tied to existing curricular outcomes. Background documents on the aims/goals/importance of media literacy and food marketing are necessary, as is the availability of presentation materials (e.g., fact sheets, slide-decks, handouts), tangible tools for activities (e.g., food ads, packaged foods, whole foods) and evaluation methods (e.g., post-intervention surveys, homework assignments).

For *parents/caregivers*, to facilitate children's continued practice of applying media literacy at home, fact sheets with activities for analysing persuasive food marketing messages are useful, along with food label-reading activities (such as ensuring that front-of-package visuals on food packages do not trump product nutrition when evaluating healthfulness), and discussion points for grocery store shopping. Given that caregivers make the food purchasing/preparation decisions, such support can contribute to an improved food environment for children–although we recognise that even the most robust media literacy skills cannot address the structural barriers (such as food insecurity, access to fresh, whole foods, etc.) that may profoundly impact food choices.

Recommended best practices for *evaluating* media literacy and food marketing programming include *using mixed methods* to provide a nuanced understanding of program results. This may begin with quantitative methods (i.e., pre-post tests, surveys) to observe knowledge acquisition at the level of the individual child, including their recognition and understanding of the overall persuasive intent and specific techniques of persuasion within the advertising message. Qualitative methods, including focus groups and open-ended questions (on post-tests and exit surveys) allow for a deeper understanding of children's perspectives around food/brand attitudes and preferences. Qualitative methods can also enable researchers and practitioners of media literacy to evaluate how effectively children apply knowledge about food promotion to new examples or contexts (11). Beyond this, *tracking program reach* (number of uses) and post-post-interventions (follow-up at 6 months, or 1 year post-program) help in measuring longer-term impacts.

Throughout the BBE meeting, stakeholders emphasised both the complexity of measuring children's critical media literacy skills and that developing such skills is not a “one-off” process. Facilitating children's *ongoing media literacy development* involves also inquiring into the capabilities and confidence of teachers and parents/caregivers so that adequate support can be provided to them. Doing so can help foster the community required to encourage continued critical reflection, alongside the critical skills children need to navigate a complex food environment.

Our perspective on “best practices” is intended as a starting point for discussion. Yet the relevance of these strategies extends beyond food promotion and evaluation, and are applicable more broadly to researchers, practitioners and educators promoting critical skills in relation to health. We do not advocate for media literacy as a *substitute* for effective government policy to protect children from the negative impacts of food marketing. Instead, we view media literacy on food marketing as an essential life skill that will support children's health now and across their lifetimes.

## Data Availability Statement

The datasets presented in this article are not readily available because the qualitative data is reported in the manuscript. Requests to access the datasets should be directed to charlene.elliott@ucalgary.ca.

## Author Contributions

CE conceived the commentary topic. CE, ET, and KE drafted the manuscript. All authors attended the stakeholder meeting and contributed content, reviewed the draft manuscript and provided feedback, contributed to the article, and approved the submitted version.

## Funding

This project was generously supported with funding from a SSHRC Connection Grant (611-2020-0221), the Helderleigh Foundation, the University of Calgary Faculty of Arts, and the CIHR Canada Research Chairs program.

## Conflict of Interest

The authors declare that the research was conducted in the absence of any commercial or financial relationships that could be construed as a potential conflict of interest.

## Publisher's Note

All claims expressed in this article are solely those of the authors and do not necessarily represent those of their affiliated organizations, or those of the publisher, the editors and the reviewers. Any product that may be evaluated in this article, or claim that may be made by its manufacturer, is not guaranteed or endorsed by the publisher.

## Footnotes

^1^ https://www.rainbowplate.com

^2^ www.growingchefsontario.ca

^3^ www.sixbysixteen.me

## References

[1] World Health Organization (WHO). Food Marketing Exposure and Power and Their Associations With Food-Related Attitudes, Beliefs and Behaviours: A Narrative Review. (2022). Available online at: https://apps.who.int/iris/handle/10665/351521 (accessed April 26, 2022).

[2] Smith R Kelly B Yeatman H Boyland E. Food marketing influences children's attitudes, preferences and consumption: a systematic critical review. Nutrients. (2019) 11:875. 10.3390/nu11040875 31003489 PMC6520952

[3] Heart & Stroke Foundation. The Kids are Not Alright: How the Food Beverage Industry is Marketing Our Children Youth to Death. (2017). Available online at: https://www.heartandstroke.ca/-/media/pdf-files/canada/2017-heart-month/heartandstroke-reportonhealth2017.ashx (accessed April 26, 2022).

[4] Potvin Kent M Pauzé E Roy E de Billy N Czoli C. Children and adolescents' exposure to food and beverage marketing in social media apps. Pediatr Obes. (2019) 14:e12508. 10.1111/ijpo.12508 30690924 PMC6590224

[5] Potvin K Velazquez CE Pauzé E Cheng-Boivin O Berfeld N. Food and beverage marketing in primary and secondary schools in Canada. BMC Public Health. (2019) 19:114. 10.1186/s12889-019-6441-x 30691422 PMC6348619

[6] Velazquez CE Daepp MIG Black JL. Assessing exposure to food and beverage advertisements surrounding schools in Vancouver, BC. Health Place. (2019) 58:102066. 10.1016/j.healthplace.2018.12.007 30639201

[7] Fernandez MA Desroches S Marquis M Lebel A Turcotte M Provencher V. Promoting meal planning through mass media: awareness of a nutrition campaign among Canadian parents. Public Health Nutr. (2019) 22:3349–59. 10.1017/S1368980019002957 31663493 PMC10260515

[8] Begley A Paynter E Dhaliwal SS. Evaluation tool development for food literacy programs. Nutrients. (2018) 10:1617. 10.3390/nu10111617 30400130 PMC6267114

[9] Livingstone S. The Changing Nature Uses of Media Literacy. (2003). Available online at: http://eprints.lse.ac.uk/13476/ (accessed April 26, 2022).

[10] National Association for Media Literacy Education. Media Literacy Defined. (2022). Available online at: https://namle.net/resources/media-literacy-defined/ (accessed February 1, 2022).

[11] Truman E Elliott C. Health-promoting skills for children: evaluating the influence of a media literacy and food marketing intervention. Health Educ J. (2019) 79:431–45. 10.1177/0017896919889647

[12] World Health Organization. A Framework for Implementing the Set of Recommendations on the Marketing of Foods and Non-alcoholic Beverages to Children. (2012). Available online at: http://apps.who.int/iris/bitstream/10665/80148/1/9789241503242_eng.pdf?ua=1 (accessed March 23, 2022).

[13] Herrewijn L De Jans S Hudders L Cauberghe V. Leveling up advertising literacy! Investigating the cognitive and motivational effectiveness of a digital game for learning aimed at improving children's advertising literacy. Electron Commer Res Appl. (2021) 4:101036. 10.1016/j.elerap.2021.101036

[14] Nelson M R Powell R Ferguson GM Tian K. Using subvertising to build families' persuasion knowledge in Jamaica. J Advert. (2020) 49:477–94. 10.1080/00913367.2020.1783725

